# Inverse planning optimization for hybrid prostate permanent‐seed implant brachytherapy plans using two source strengths

**DOI:** 10.1120/jacmp.v11i3.3096

**Published:** 2010-06-03

**Authors:** J. Adam M. Cunha, Barby Pickett, Jean Pouliot

**Affiliations:** ^1^ Physics Division, Department of Radiation Oncology The University of California San Francisco CA 94115 USA

**Keywords:** brachytherapy, prostate cancer, hybrid plans, prostate permanent‐seed implant (PPI), optimization, IPSA

## Abstract

The purpose is to demonstrate the ability to generate clinically acceptable prostate permanent seed implant plans using two seed types which are identical except for their activity. The IPSA inverse planning algorithms were modified to include multiple dose matrices for the calculation of dose from different sources, and a selection algorithm was implemented to allow for the swapping of source type at any given source position. Five previously treated patients with a range of prostate volumes from $20–48\;{\text{cm}}^{3}$ were re‐optimized under two hybrid scenarios: (1) using 0.32 and $0.51\;\text{mGy}\cdot m^{2}/h$ ${\;}^{125}I$, and (2) using 0.64 and $0.76\;\text{mGy}\cdot m^{2}/h$ ${\;}^{125}I$. Isodose lines were generated and dosimetric indices, $V_{150}^{\text{Prostate}}$, $D_{90}^{\text{Prostate}}$, $V_{150}^{\text{Urethra}}$, $V_{125}^{\text{Urethra}}$, $V_{120}^{\text{Urethra}}$, $V_{100}^{\text{Urethra}}$, and $D_{10}^{\text{Urethra}}$ were calculated. The algorithm allows for the generation of single‐isotope, multi‐activity hybrid brachytherapy plans. By dealing with only one radionuclide, but of different activity, the biology is unchanged from a standard plan. All $V_{100}^{\text{Prostate}}$ were within 2.3 percentage points for every plan and always above the clinically desirable 95%. All $V_{150}^{\text{Urethra}}$ were identically zero, and $V_{120}^{\text{Urethra}}$ is always below the clinically acceptable value of $1.0\;{\text{cm}}^{3}$. Clinical optimization times for the hybrid plans are still under one minute, for most cases. It is possible to generate clinically advantageous brachytherapy plans (i.e. obtain the same quality dose distribution as a standard single‐activity plan) while incorporating leftover seeds from a previous patient treatment. This method will allow a clinic to continue to provide excellent patient care, but at a reduced cost. Multi‐activity hybrid plans were equal in quality (as measured by the standard dosimetric indices) to plans with seeds of a single activity. Despite the expanded search space, optimization times for these studies were still under two minutes on a modern day laptop and can be reduced to below one minute in a clinical setting. With the typical cost of a set of PPI seeds on the order of thousands of dollars, it is possible to reduce the cost of brachytherapy treatments by allowing for easier use of seeds left over from a previous patient or unused due to a cancelled treatment.

PACS number: 87.55.D‐, 87.55.Kd, 87.55.ne

## I. INTRODUCTION

Current permanent prostate implant brachytherapy (PPI) dose plans use one type of radioactive seed per treatment plan, all with the same activity. Radioactive seeds must be ordered from a vendor and used before the radioactivity dissipates. The half‐life of one of the more common radionuclides, ${\;}^{125}I$, is approximately 60 days; therefore, if the seeds are not used within one week, the activity will have decreased by approximately 10%. For seeds like ${\;}^{103}\text{Pd}$ and ${\;}^{131}\text{Cs}$, with half lives of 17 and 10 days respectively, the decrease is even greater (Table 1). Thus, if a treatment is missed, seeds — and therefore money — are wasted. These seeds are not entirely unusable and may be used for a different patient by generating a plan using the partially‐decayed activity^†^; however, this can only be done if there are enough seeds for an entire plan.

**Table 1:** Half‐lives for commonly‐used PPI seeds: $0.76\;\text{mGy}\cdot m^{2}/h(0.6\;\text{mCi})$ ${\;}^{125}I=0.65\;\text{mGy}\cdot m^{2}/h(0.51\;\text{mCi})$ ${\;}^{125}I$ after two weeks.

*Seed Type*	*Half Life*	*Activity after*	
		*7 days*	*14 days*
Iodine‐125	59.9 days	92%	85%
Palladium‐103	17.0 days	75%	57%
Cesium‐131	9.7 days	62%	38%

In addition, it has been reported that it is important to account for differences in the seeds supplied by different manufacturers when generating treatment plans and calculating dosimetry. Identical plans using the same radionuclide but from different vendors will have different dosimetry due to different dose rate constants, anisotropy factors, and radial dose functions of each vendor's seeds.^(^ ^1^ ^,^ ^2^ ^,^ ^3^ ^)^

There has been recent interest in generating dose plans with two different radionuclides. In their 2002 paper on the feasibility of ${\;}^{192}\text{Ir}$ seeds for PPI, Glasgow et al.^(^ ^4^ ^)^ proposed that combinations of ${\;}^{192}\text{Ir}$ and ${\;}^{125}I$ seeds can provide adequate coverage of the prostate and sparing of the organs at risk. In this case, the argument is that ${\;}^{192}\text{Ir}$ and ${\;}^{125}I$ have similar half lives (73.83 days verses 59.40 days) and similar absorbed doses delivered in the permanent implant. Therefore, the radiobiological effects of using two different radionuclides is negligible for cases which used up to $40\;\text{mGy}\cdot m^{2}/h(10\;\text{mCi})$ ${\;}^{192}\text{Ir}$. In 2007, Chaswal et al.^(^ ^5^ ^)^ expanded on the forward planning‐based work of Glasgow and used a Greedy Heuristic algorithm to optimize the treatment plans. They showed that there can be dosimetric and trauma‐reducing benefits to using plans with a combination of ${\;}^{192}\text{Ir}$ and ${\;}^{125}I$. These studies are promising in that they show a clinical benefit can be derived from including more than one radionuclide in a given brachytherapy plan. However, the results are dependent on knowing and understanding the biology of the superposition of two different radiation delivery mechanisms.

There has been progress made recently in our understanding of the biological responses to different dose delivery characteristics of different radionuclides. In fact, some recent studies have shown no significant difference between biochemical failure and control as a function of biological equivalent dose (BED), equivalent uniform dose (EUD), or tumor control probability (TCP) when comparing plans using ${\;}^{125}I$ and ${\;}^{103}\text{Pd}$.^(^ ^6^ ^)^ However, even setting aside the uncertainty in CT‐reconstructed target volumes, there is a lack of consensus about the actual value of the biological parameters that govern the definition of the BED, EUD, and TCP, with values of α/β differing by more than a factor of 2.^(^ ^7^ ^)^ The uncertainty in the models that predict the biological effect of the specific characteristics naturally propagates to the dosimetry of brachytherapy plans that incorporate multiple radionuclides.

However, assuming the uncertainty in the biology is acceptable, there does appear to be a benefit to using multiple types of seeds (each with a different dosimetry) in one brachytherapy plan.^(^ ^4^ ^,^ ^5^ ^)^ In this work, we aim to avoid the biology‐based uncertainties attached to the use of two different radionuclides. It would be beneficial to be able to generate clinically‐acceptable hybrid plans that incorporate seeds of the same radionuclide, but with different characteristics — be they different activity or slightly different dosimetric geometry. In contrast to plans using multiple radionuclides, single radionuclide hybrid plans are free from the biological uncertainties associated with the efficacy combining characteristically different radiation.

We present an algorithm that allows for the generation of single‐isotope, multi‐activity hybrid brachytherapy plans. By dealing with only one radionuclide, but of different activity, it is possible to generate clinically advantageous brachytherapy plans: i.e. obtain the same quality dose distribution as a standard single‐activity plan while incorporating left over seeds from a previous patient treatment. This method will allow a clinic to continue to provide excellent patient care, but at a reduced cost.

## II. MATERIALS AND METHODS

### A. Code development rational

The algorithms developed for this work augment the IPSA inverse planning algorithms.^(^ ^8^ ^,^ ^9^ ^,^ ^10^ ^,^ ^11^ ^)^ While the core optimization engine remains unchanged, the code was modified in three ways: (1) more than one radionuclide source can be specified using the TG‐43 formalism;^(^ ^12^ ^)^ (2) the system for evaluating the dose delivered to the target and surrounding organs was expanded to incorporate two separate radionuclide dose profiles; and (3) the optimization engine can switch the seed type at each source position at specific moments during the iteration process.

As in the original algorithm, to determine the dose, $D_{j}$, delivered to unit volume (voxel) *j* of an organ, the contributions from the $i^{\text{th}}$ source position, $D_{\mathrm{ij}}$, are summed:

(1) $$D_{j}=\sum_{i}^{N_{p}}D_{ij};$$

$N_{p}$ is the number of source positions. The total dose, *D*, to an organ is the sum of the dose at each voxel within that organ, $N_{v}$ is the number of voxels in the organ.

(2) $$D=\sum_{i}^{N_{v}}D_{j}.$$

However, in the new algorithm, the dose matrix ($D_{\mathrm{ij}}$) used depends on which seed is present at position *i*. During the initialization phase of the optimization process, the algorithm computes a separate dose matrix for each seed type. Then an initial configuration of seeds is placed in the target. This configuration is random, but the user has the ability to specify a desired number of each seed type. The user also has the ability to permanently set the seed type at any source position.

The algorithm then proceeds to the iteration phase, during which the search space is probed by placing or removing seeds of either type at randomly selected source positions. The new configuration is then (1) evaluated to determine the value of the objective function^‡^, (2) compared to the best configuration yet attained, and (3) kept or rejected based on the result of No. 2.

In order to maximize clinical relevance, the optimization parameters were chosen from the class solution developed in Lessard et al.^(^ ^8^ ^)^ and Pouliot.^(^ ^13^ ^)^ This class solution was shown to mimic an experienced dosimetrist by consistently producing dose plans equivalent (as measured by common dosimetric indices) to those created by a dosimetrist in our clinic. This allows for better isolation of the variables which control the use of partial‐activity seeds in the optimization. Two limits on the number of needles allowed per plan are included: a soft penalty and an upper limit. The soft penalty is a weight multiplied by the total number of needles and then added into the objective function of the optimization. The upper limit restricts the number of needles allowed in one implant. Throughout the studies in this paper, the upper limit is set to 30. This is the limit our clinic imposes, since our clinical experience has led to the conventional wisdom that, as the number of needles in one implant approaches 30, the complications due to edema, erectile dysfunction and other trauma begin to have unacceptably deleterious effects on the post‐treatment quality of life of the patient. On average our clinic uses 25 needles per treatment plan for a $40\;{\text{cm}}^{3}$ prostate gland.

### B. Dosimetric analysis

The quality of the plans generated in this analysis will be graded using the standard dosimetric indices developed by the American Association of Physicists in Medicine (AAPM), the American Brachytherapy Society (ABS), and our clinic. For brachytherapy treatment of prostate cancer, Stock et al.^(^ ^14^ ^)^ have demonstrated that the minimum dose delivered to at least 90% of the gland ($D_{90}^{\text{Prostate}}$) had a critical impact on the subsequent risk of prostate‐specific antigen‐diagnosed recurrence. The American Brachytherapy Society has published general guidelines for different anatomical sites^(^ ^15^ ^)^ that consist of a set of dose limit specifications. Our clinic incorporates these recommendations and imposes even stricter limits for the target coverage and urethra dose,^(^ ^8^ ^)^ which have yielded reproducible results: mature five‐year biochemical control rates of 96% (median follow‐up 63 months) reported for 118 consecutive patients.^(^ ^16^ ^)^ Plan quality was evaluated by the UCSF clinical physicist with experience generating over 1500 plans. For the purposes of this study, the single‐activity plans were considered the control sample against which the dosimetry of the multiple‐activity plans was judged. The validity of the standard (single‐activity) plans was examined and established in previous work.^(^ ^8^ ^)^

#### B.1 Target dose coverage

The target coverage was compared across plans by examining volume of the prostate receiving at least the prescription dose ($V_{100}^{\text{Prostate}}$), at least 150% of the prescription dose ($V_{150}^{\text{Prostate}}$), and the minimum dose that covers 90% of the prostate ($D_{90}^{\text{Prostate}}$). In general, we strive to achieve $V_{100}^{\text{Prostate}}$ >95% and $V_{150}^{\text{Prostate}}$ <65%. A $V_{100}^{\text{Prostate}}$ above 95% ensures complete coverage of the target, but will encourage a value of $D_{90}^{\text{Prostate}}$ 10%–20% above the prescription dose. Because this is a not a post‐implant dosimetry analysis, but rather a pre‐implant dosimetry analysis, it is important to see the $D_{90}^{\text{Prostate}}$ higher than the prescription dose by approximately this amount,^(^ ^2^ ^,^ ^3^ ^)^ since the dosimetry generally sees a decrease in post‐implant $D_{90}^{\text{Prostate}}$ with respect to the pre‐implant $D_{90}^{\text{Prostate}}$.

#### B.2 Urethral dosimetry

One of the most common side effects of prostate brachytherapy (urethral stricture) is caused by excessive dose delivered to the urethra. The urethral volume receiving at least 100%, 120%, 125%, and 150% of the presection dose ($V_{100}^{\text{Urethra}}$, $V_{120}^{\text{Urethra}}$, $V_{125}^{\text{Urethra}}$, and $V_{150}^{\text{Urethra}}$, respectively) as well as the minimum dose delivered to 10% of the urethra ($D_{10}^{\text{Urethra}}$), can be used to monitor the dose delivered to the urethra. To adequately protect the urethra, we strive to obtain $V_{125}^{\text{Urethra}}$ $<1.0\;{\text{cm}}^{3}$ and $V_{120}^{\text{Urethra}}$ $<1.0\;{\text{cm}}^{3}$.

### C. Procedure

Five previously treated patients with a range of prostate volumes from 20 to $48\;{\text{cm}}^{3}$ were chosen and reoptimized using the hybrid‐activity optimization. Different clinics use different seed activities — 0.3–0.5 mCi and up to 0.7 mCi are common.^(^ ^17^ ^)^ Since our clinic uses 0.39 mCi, we performed this work using two different sets of seed activity. Four studies were organized into two scenarios. Of course the work presented here should not be considered indicative of the clinical work done at any institution; since many factors go into generating plans, these studies were kept as general as possible. In Scenario I, we generate hybrid plans using 0.32 and $0.51\;\text{mGy}\cdot m^{2}/h{\left(0.25\;\text{and}\;0.40\;\text{mCi}\right)}^{125}I$ seeds $\left(A_{0}/A_{0}=62.5\%\right)$. In Scenario II, we generate hybrid plans using 0.64 and $0.76\;\text{mGy}.m^{2}/h{\left(0.50\;\text{and}\;0.60\;\text{mCi}\right)}^{125}I$ seeds $A/A_{0}=83.3\%$. Table 2 outlines the parameters of the studies performed.

**Table 2:** A summary of the studies performed.

*Study*	*Air Kerma Rate* ${\;}^{125}I(U)$	*Per‐needle Penalty*	*Results Table*
Scenario I; Study 1	0.32 & 0.51	On	Table 3
Scenario I; Study 2	0.32 & 0.51	Off	Table 4
Scenario II; Study 1	0.64 & 0.76	On	Table 5
Scenario II; Study 2	0.64 & 0.76	Off	Table 6

For each study five plan types were generated

**Table 3:** Scenario I; Study 1. Data showing five separate plans generated for each patient case. Plans were generated with: (1) only one seed activity available to the optimization, 0.51 U ${\;}^{125}I$; (2) a requested 30 seeds of 0.32 U ${\;}^{125}I$ activity plus any number of seeds of 0.51 U ${\;}^{125}I$; (3) a requested 60 seeds of 0.32 U ${\;}^{125}I$ plus any number of seeds of 0.51 U ${\;}^{125}I$; (4) only 0.32 U ${\;}^{125}I$ seeds available to the optimization; (5) an unrestricted hybrid mix of 0.32 and 0.51 U ${\;}^{125}I$. Per‐needle penalty is on.

*Target Volume (cm^3^)*	*Plan Type*	*# of Needles*	*Total Seeds*	*Low Act. #(%)*	$V_{100}^{\mathrm{Prostate}}$ *(%)*	$V_{150}^{\mathrm{Prostate}}$ *(%)*	$D_{90}^{\mathrm{Prostate}}$ *(Gy)*	$D_{100}^{\mathrm{Urethra}}$ *(cm^3^)*	$V_{120}^{\mathrm{Urethra}}$ *(cm^3^)*	$V_{125}^{\mathrm{Urethra}}$ *(cm^3^)*	$D_{10}^{\mathrm{Urethra}}$ *(Gy)*
48	Only 0.51 U	26	85	0(0)	96.9	64.0	178	0.6	0.4	0.1	184
48	30 0.32 U	29	94	28(30)	99.2	56.4	174	0.6	0.1	0.1	184
48	60 0.32 U	29	105	57(54)	98.2	59.6	178	0.6	0.2	0.1	186
48	Only 0.32 U	30	130	130(100)	98.6	57.2	178	0.6	0.3	0.1	186
48	Pure hybrid	29	94	24(26)	98.2	57.3	180	0.6	0.1	0.0	177
47	Only 0.51 U	23	89	0(0)	99.4	61.4	179	0.8	0.1	0.0	174
47	30 0.32 U	26	100	30(30)	99.6	59.5	173	0.8	0.1	0.0	171
47	60 0.32 U	26	109	58(53)	99.2	59.3	175	0.8	0.1	0.0	175
47	Only 0.32 U	30	137	137(100)	98.8	61.9	175	0.9	0.1	0.0	175
47	Pure hybrid	25	100	32(32)	97.6	60.2	173	0.8	0.1	0.0	173
36	Only 0.51 U	28	73	0(0)	96.6	63.9	173	0.5	0.1	0.0	177
36	30 0.32 U	30	85	30(35)	97.3	63.7	181	0.5	0.1	0.0	177
36	60 0.32 U	30	94	58(62)	98.2	62.0	176	0.5	0.0	0.0	173
36	Only 0.32 U	39	111	111(100)	97.6	59.8	176	0.6	0.0	0.0	173
36	Pure hybrid	28	82	25(30)	97.1	61.6	178	0.5	0.1	0.0	178
29	Only 0.51 U	22	63	0(0)	98.2	62.7	178	0.9	0.2	0.0	178
29	30 0.32 U	25	74	29(39)	98.0	61.5	180	0.8	0.2	0.0	177
29	60 0.32 U	30	85	59(69)	97.9	61.6	176	0.8	0.2	0.0	177
29	Only 0.32 U	30	97	97(100)	97.4	56.7	176	0.8	0.0	0.0	177
29	Pure hybrid	25	71	22(31)	97.8	58.7	177	0.9	0.0	0.0	174
20	Only 0.51 U	18	47	0(0)	96.9	64.7	175	0.4	0.2	0.2	189
20	30 0.32 U	18	57	29(51)	96.6	62.3	174	0.4	0.2	0.1	183
20	60 0.32 U	22	66	57(86)	96.7	64.1	173	0.4	0.2	0.1	188
20	Only 0.32 U	22	70	70(100)	97.3	62.7	173	0.4	0.2	0.1	188
20	Pure hybrid	19	51	12(24)	96.7	60.4	175	0.4	0.2	0.1	184

**Table 4:** Scenario I; Study 2. Data showing five separate plans generated for each patient case. Plans were generated with: (1) only one seed activity available to the optimization, 0.51 U ${\;}^{125}I$; (2) a requested 30 seeds of 0.32 U ${\;}^{125}I$ activity plus any number of seeds of 0.51 U ${\;}^{125}I$; (3) a requested 60 seeds of 0.32 U ${\;}^{125}I$ plus any number of seeds of 0.51 U ${\;}^{125}I$; (4) only one seed activity available, 0.32 U ${\;}^{125}I$; (5) unrestricted hybrid mix of 0.32 U and 0.51 U ${\;}^{125}I$. Per‐needle penalty is off.

*Target Volume (cm^3^)*	*Plan Type*	*# of Needles*	*Total Seeds*	*Low Act. #(%)*	$V_{100}^{\mathrm{Prostate}}$ *(%)*	$V_{150}^{\mathrm{Prostate}}$ *(%)*	$D_{90}^{\mathrm{Prostate}}$ *(Gy)*	$D_{100}^{\mathrm{Urethra}}$ *(cm^3^)*	$V_{120}^{\mathrm{Urethra}}$ *(cm^3^)*	$V_{125}^{\mathrm{Urethra}}$ *(cm^3^)*	$D_{10}^{\mathrm{Urethra}}$ *(Gy)*
48	Only 0.51 U	30	86	0(0)	97.9	63.9	179	0.6	0.3	0.1	183
48	30 0.32 U	30	97	30(31)	99.0	56.2	181	0.6	0.1	0.0	176
48	60 0.32 U	30	105	58(55)	99.0	58.6	179	0.6	0.2	0.1	181
48	Only 0.32 U	30	129	129(100)	99.1	54.1	179	0.6	0.2	0.0	181
48	Pure hybrid	30	96	29(30)	99.3	56.5	180	0.6	0.1	0.0	178
47	Only 0.51 U	29	88	0(0)	99.4	62.4	178	0.8	0.1	0.0	176
47	30 0.32 U	30	100	30(30)	99.6	60.5	173	0.8	0.0	0.0	173
47	60 0.32 U	30	110	60(55)	98.5	58.8	171	0.8	0.1	0.0	175
47	Only 0.32 U	30	136	136(100)	99.3	61.0	171	0.9	0.1	0.0	175
47	Pure hybrid	29	100	29(29)	99.0	57.1	176	0.8	0.1	0.0	174
36	Only 0.51 U	30	74	0(0)	96.9	63.5	173	0.6	0.0	0.0	174
36	30 0.32 U	30	85	29(34)	97.5	61.4	179	0.5	0.1	0.0	176
36	60 0.32 U	30	95	59(62)	98.2	61.2	176	0.6	0.0	0.0	174
36	Only 0.32 U	30	111	111(100)	98.2	59.3	176	0.6	0.1	0.0	174
36	Pure hybrid	30	85	31(36)	96.7	60.8	178	0.5	0.1	0.0	175
29	Only 0.51 U	29	64	0(0)	97.7	62.7	178	0.8	0.1	0.0	176
29	30 0.32 U	30	74	29(39)	97.9	59.6	179	0.8	0.1	0.0	173
29	60 0.32 U	30	85	60(71)	98.9	62.3	175	0.9	0.1	0.0	173
29	Only 0.32 U	30	96	96(100)	97.7	59.0	175	0.9	0.0	0.0	173
29	Pure hybrid	29	75	31(41)	97.9	59.4	176	0.8	0.0	0.0	172
20	Only 0.51 U	19	47	0(0)	97.6	66.6	182	0.4	0.2	0.2	194
20	30 0.32 U	22	68	30(52)	97.6	63.7	179	0.4	0.2	0.1	183
20	60 0.32 U	24	66	57(86)	96.9	64.7	175	0.4	0.2	0.2	189
20	Only 0.32 U	25	71	71(100)	96.9	63.8	175	0.4	0.2	0.1	189
20	Pure hybrid	21	53	16(30)	96.4	62.1	177	0.4	0.2	0.0	180

**Table 5:** Scenario II; Study 1. Data showing five separate plans generated for each patient case. Plans were generated with: (1) only one seed activity available to the optimization, 0.76 U ${\;}^{125}I$; (2) a requested 30 seeds of 0.64 U ${\;}^{125}I$ activity plus any number of seeds of 0.60 mCi ${\;}^{125}I$; (3) a requested 60 seeds of 0.64 U ${\;}^{125}I$ plus any number of seeds of 0.76 U ${\;}^{125}I$; (4) only 0.64 U ${\;}^{125}I$ seeds available to the optimization; (5) an unrestricted hybrid mix of 0.64 and 0.76 U ${\;}^{125}I$. Per‐needle penalty is on.

*Target Volume (cm^3^)*	*Plan Type*	*# of Needles*	*Total Seeds*	*Low Act. #(%)*	$V_{100}^{\mathrm{Prostate}}$ *(%)*	$V_{150}^{\mathrm{Prostate}}$ *(%)*	$D_{90}^{\mathrm{Prostate}}$ *(Gy)*	$D_{100}^{\mathrm{Urethra}}$ *(cm^3^)*	$V_{120}^{\mathrm{Urethra}}$ *(cm^3^)*	$V_{125}^{\mathrm{Urethra}}$ *(cm^3^)*	$D_{10}^{\mathrm{Urethra}}$ *(Gy)*
48	Only 0.76 U	22	58	0(0)	98.6	50.3	174	0.6	0.2	0.0	179
48	30 0.64 U	23	62	29(47)	99.3	50.9	172	0.6	0.1	0.1	181
48	60 0.64 U	23	68	60(88)	98.4	53.8	178	0.7	0.2	0.1	183
48	Only 0.64 U	25	69	69(100)	99.6	57.0	178	0.7	0.1	0.1	183
48	Pure hybrid	23	63	33(52)	99.1	56.0	177	0.6	0.1	0.1	181
47	Only 0.76 U	28	61	0(0)	98.7	59.9	169	0.9	0.1	0.0	173
47	30 0.64 U	30	65	30(46)	98.0	56.8	175	0.8	0.1	0.0	175
47	60 0.64 U	29	70	59(84)	98.5	62.4	175	0.8	0.1	0.0	176
47	Only 0.64 U	28	72	72(100)	96.9	56.9	175	0.8	0.1	0.0	176
47	Pure hybrid	28	65	33(51)	98.2	57.1	171	0.8	0.0	0.0	172
36	Only 0.76 U	28	51	0(0)	96.9	57.7	176	0.5	0.2	0.2	186
36	30 0.64 U	29	55	29(53)	95.7	56.4	178	0.5	0.1	0.0	178
36	60 0.64 U	28	60	59(98)	96.1	60.1	176	0.5	0.1	0.0	175
36	Only 0.64 U	28	60	60(100)	97.0	58.4	176	0.5	0.2	0.0	175
36	Pure hybrid	30	55	28(51)	96.3	58.0	180	0.5	0.2	0.0	179
29	Only 0.76 U	26	43	0(0)	97.3	57.6	177	0.8	0.2	0.0	177
29	30 0.64 U	25	48	29(60)	96.5	53.9	174	0.7	0.0	0.0	171
29	60 0.64 U	26	52	52(100)	97.6	58.5	179	0.7	0.1	0.0	176
29	Only 0.64 U	28	52	52(100)	97.0	57.1	179	0.9	0.0	0.0	176
29	Pure hybrid	27	47	19(40)	97.9	53.3	179	0.8	0.0	0.0	172
20	Only 0.76 U	17	32	0(0)	94.7	62.0	172	0.4	0.2	0.1	185
20	30 0.64 U	18	37	29(78)	97.3	58.8	173	0.4	0.2	0.0	181
20	60 0.64 U	18	39	39(100)	94.7	62.0	172	0.4	0.2	0.1	185
20	Only 0.64 U	18	39	39(100)	93.7	59.6	172	0.4	0.2	0.1	185
20	Pure hybrid	18	35	17(49)	95.1	56.1	171	0.4	0.2	0.0	181

**Table 6:** Scenario II; Study 2. Study of 0.635 U vs. 0.6 mCi and hybrid plans. Data showing five separate plans generated for each patient case – Prostate dosimetric indices. Plans were generated with: (1) only one seed activity available to the optimization, 0.76 U ${\;}^{125}I$; (2) a requested 30 seeds of 0.64 U ${\;}^{125}I$ activity plus any number of seeds of 0.60 mCi ${\;}^{125}I$; (3) a requested 60 seeds of 0.64 U ${\;}^{125}I$ plus any number of seeds of 0.76 U ${\;}^{125}I$; (4) only 0.64 U ${\;}^{125}I$ seeds available to the optimization; (5) an unrestricted hybrid mix of 0.64 and 0.76 U ${\;}^{125}I$. Per‐needle penalty off.

*Target Volume (cm^3^)*	*Plan Type*	*# of Needles*	*Total Seeds*	*Low Act. #(%)*	$V_{100}^{\mathrm{Prostate}}$ *(%)*	$V_{150}^{\mathrm{Prostate}}$ *(%)*	$D_{90}^{\mathrm{Prostate}}$ *(Gy)*	$D_{100}^{\mathrm{Urethra}}$ *(cm^3^)*	$V_{120}^{\mathrm{Urethra}}$ *(cm^3^)*	$V_{125}^{\mathrm{Urethra}}$ *(cm^3^)*	$D_{10}^{\mathrm{Urethra}}$ *(Gy)*
48	Only 0.76 U	30	57	0(0)	99.3	52.6	177	0.6	0.1	0.0	181
48	30 0.64 U	30	62	29(47)	99.3	46.2	173	0.6	0.1	0.1	181
48	60 0.64 U	30	68	60(88)	99.0	56.7	180	0.6	0.1	0.0	177
48	Only 0.64 U	30	69	69(100)	99.3	50.1	180	0.6	0.1	0.1	177
48	Mix	30	64	36(56)	99.5	47.6	174	0.6	0.1	0.1	182
47	Only 0.76 U	28	62	0(0)	98.0	58.7	174	0.8	0.2	0.1	176
47	30 0.64 U	30	66	30(45)	98.1	57.2	165	0.8	0.1	0.0	175
47	60 0.64 U	29	70	59(84)	98.0	55.5	167	0.9	0.1	0.0	175
47	Only 0.64 U	28	73	73(100)	98.4	52.8	167	0.8	0.1	0.0	175
47	Mix	28	65	29(45)	97.9	55.7	171	0.9	0.1	0.0	173
36	Only 0.76 U	28	51	0(0)	96.2	57.3	176	0.5	0.1	0.0	180
36	30 0.64 U	29	55	29(53)	97.3	57.4	175	0.5	0.2	0.1	184
36	60 0.64 U	28	60	58(97)	96.4	61.7	176	0.5	0.1	0.0	180
36	Only 0.64 U	28	60	60(100)	96.5	56.5	176	0.5	0.2	0.1	180
36	Mix	30	55	26(47)	96.7	59.8	180	0.5	0.1	0.0	180
29	Only 0.76 U	26	44	0(0)	96.0	56.8	170	0.4	0.2	0.0	177
29	30 0.64 U	25	49	29(59)	96.3	60.7	170	0.4	0.2	0.1	183
29	60 0.64 U	26	52	52(100)	96.0	56.8	170	0.4	0.2	0.0	177
29	Only 0.64 U	28	53	53(100)	98.3	64.6	170	0.4	0.2	0.1	177
29	Mix	27	47	20(43)	96.7	60.9	168	0.4	0.2	0.1	182
20	Only 0.76 U	17	33	0(0)	98.3	57.4	181	0.8	0.1	0.0	176
20	30 0.64 U	18	38	30(79)	96.7	58.5	181	0.8	0.0	0.0	173
20	60 0.64 U	18	38	38(100)	98.1	58.7	176	0.8	0.0	0.0	174
20	Only 0.64 U	18	38	38(100)	97.3	54.2	176	0.8	0.1	0.0	174
20	Mix	18	36	18(50)	97.0	60.0	181	0.8	0.1	0.0	176

For each study, five plan types were generated with a prescription dose of 144 Gy:

- Uni‐activity–full activity: Only one seed type (the full activity) is available to theoptimization
- 30 partial activity: The optimization is requested to use 30 of the partial activity seeds plus any number of the full activity seeds
- 60 partial activity: The optimization is requested to use 60 of the partial activity seeds plus any number of the full activity seeds
- Uni‐activity–partial activity: Only one seed (the partial activity) available to the optimization
- Unrestricted mix (pure hybrid): Allow the optimization to employ any number of each seed type

The soft needle penalty encourages the optimization to minimize the number of needles in addition to optimizing the dose distribution. For Study 1 in Scenario I and II, this needle penalty is set to the value used in our clinic. For Study 2 in Scenario I and II, this was removed and the optimization was run with no per‐needle penalty.

## III. RESULTS

**Figure 1:**
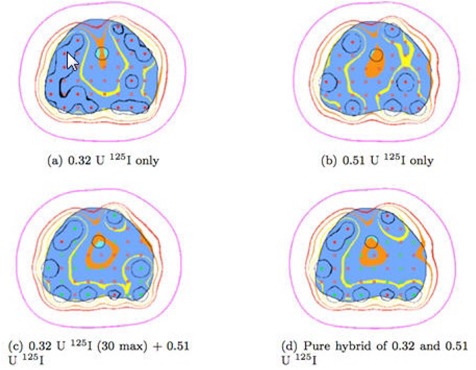
Scenario I, Study 1: Isodose lines for one slice of the $48\;{\text{cm}}^{3}$ prostrate. Isodose lines (outside in) are 50% (purple), 100% (red), 125% (orange), 150% (yellow), 150% (black).

**Figure 2:**
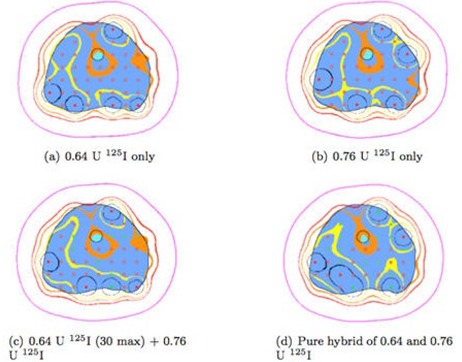
Scenario II, Study 1: Isodose lines for one slice of the $48\;{\text{cm}}^{3}$ prostate. Isodose lines (outside in) are 50% (purple), 100% (red), 125% (orange), 150% (yellow), 150% (black).

The dosimetric results for Scenario I are presented in Tables 3 and 4, and a set of isodose lines are shown in Fig. 1. The same are presented for Scenario II in Tables 5 & 6 and Fig. 2. The number of seeds of each activity is listed along with the standard dosimetric indices for the prostate ($V_{100}^{\text{Prostate}}$, $V_{150}^{\text{Prostate}}$, and $D_{90}^{\text{Prostate}}$) and for the urethra ($V_{100}^{\text{Urethra}}$, $V_{120}^{\text{Urethra}}$, $V_{125}^{\text{Urethra}}$, and $D_{10}^{\text{Urethra}}$). $V_{\text{Urethra}150}$ is identically zero for all cases and is, therefore, not listed in the Tables.

### A. Activities 0.32 U and 0.51 U

Note that for any one case in Scenario I, Study 1 (Table 3), the target volume receiving 100% of the prescribed dose ($V_{100}^{\text{Prostate}}$) is within 2.3 percentage points for every plan and always above the clinically desirable 95%. The $D_{90}^{\text{Prostate}}$ for all plans hovers around 120% of the prescription dose. This is to be expected when evaluating the preplan dosimetry as opposed to the post‐plan dosimetry.^(^ ^2^ ^,^ ^3^ ^)^

Also note that the $V_{120}^{\text{Urethra}}$ is always below the clinically acceptable value of $1.0\;{\text{cm}}^{3}$. Study 2 under this scenario shows the effect of removing the per‐needle penalty compared to Study 1, which preserves it. Note that the effect of removing the needle penalty is to increase the number of needles and obtain a slight increase in target coverage. Comparing Table 3 with Table 4 (and Table 5 with Table 6 in Scenario II) shows the effect of the per‐needle penalty. In general, this penalty allows for a decrease in the number of needles needed independent of the hybrid status of the plan. It is interesting to note that, even in the case where the per‐needle penalty is turned on (Tables 3 and 5), the plans generated with an unrestricted mix of seeds still preferred using some partial‐activity seeds. For the studies with the per‐needle penalty turned on, the number of needles recommended for each implant is consistent with the number of needles routinely used for actual implants performed at our clinic.

### B. Activities 0.64 U and 0.76 U

The results are similar in Scenario II for both Study 1 (Table 5) and Study 2 (Table 6) across all plans. It is clear from these results that it is possible to achieve clinically acceptable dose distributions with hybrid plans that incorporate different‐activity seeds. Across all four studies of each of the five patients, there is no evidence that the prostate size (which ranges from 20 to 48 cc) has an effect on the dosimetry of the hybrid plans.

Studies were performed on the geometric distribution of each seed type. The density of seeds as a function of distance from the center of mass of the prostate was examined. The prostate was divided in octants with the origin placed at the center of the prostate^∫^, and the density of seeds in each octant was evaluated. No correlation was found between seed location and seed type.

A final study was performed in order to determine whether the optimized configuration of seed types is independent of the initial configuration. The initial configuration of seed types was set to be all of one activity or the other (as opposed to randomly chosen for each seed position, as was done in the main studies). The plans were then optimized requesting an unrestricted mix of the two seed types. The final configuration was found to have no correlation to the initial configuration of seed types.

### C. CPU time

Table 7 shows the time needed to process the optimization in Scenario I Study 1 (all studies had similar results). The baseline IPSA^(^ ^8^ ^,^ ^9^ ^,^ ^10^ ^,^ ^11^ ^)^ optimization for a single‐activity varies from 51–155 s. The hybrid optimization incorporates an increase in the degrees of freedom that must be probed, so an increase in optimization time is expected. The increase in optimization time varies from 17%–35%; however, this translates into only a 16–37 s increase in real time. Thus, the optimization time is still on the order of two minutes for all cases. Our clinical protocol for the baseline optimization uses a factor of five fewer iterations than used in this study. The optimizations were stable after a two‐fold increase in the number of iterations over the clinical baseline; so the times listed here should be considered an upper limit which can be reduced without any clinically noticeable loss in plan quality by a factor of 2.

**Table 7:** Scenario I; Study 1. CPU time required for optimization using a MacBook Pro running Mac OS 10.5.5 with a 2.33 GHz Intel Core 2 Duo processor and 3 GB RAM. On average there is a 25% increase in CPU time needed for the hybrid plan optimization; however, in real time this only amounts to at most about an 26‐second increase in optimization time. This can be further reduced by a factor of two when implemented on a clinical system where fewer iterations are needed than in this proof of hypothesis study.

*Plan Type*	*CPU Time for Each Optimization*				
	$48\;{\mathrm{cm}}^{3}$	$47\;{\mathrm{cm}}^{3}$	$36\;{\mathrm{cm}}^{3}$	$29\;{\mathrm{cm}}^{3}$	$20\;{\mathrm{cm}}^{3}$
Only 0.51 U	90 s	118 s	100 s	85 s	51 s
30 0.32 U	104 s	151 s	135 s	110 s	68 s
60 0.32 U	106 s	155 s	131 s	112 s	67 s
Only 0.32 U	97 s	125 s	104 s	90 s	51 s
Pure Hybrid	100 s	144 s	122 s	102 s	61 s
Max Increase (s)	16 s	37 s	35 s	27 s	17 s
Max Increase (%)	17%	24%	35%	24%	25%

## IV. DISCUSSION

As noted in the Introduction, the effect of the radioactive decay half‐life can have an impact on the usability of seeds more than a week old. With activity reductions on the order of 40% after one week (Table 1), it is necessary to incorporate this change in activity into the planning workflow. In and of itself, this is not a problem since it is relatively simple to calculate the current activity of the seeds available and build a treatment plan accordingly. But this only works if there are enough seeds to accommodate an entire implant. If there are fewer partial‐activity seeds than is required for the implant, the partial‐activity seeds are useless; the plan must be discarded and a new plan using full‐activity seeds must be generated.

First and foremost, it is clear from Tables 3–6 that there is no degradation of plan quality when using partial activity seeds — both coverage of the target and sparing of the urethra are stable across plans. Given that plan quality is preserved, this algorithm would save both time (by eliminating the possibility that a plan with too many partial‐activity seeds must be discarded) and money (by allowing for the use of any inventory of partial‐activity seeds). Since the algorithm includes input parameters to allow the user to chose a specific number of partial activity seeds, this method will work for any number of partial activity seeds.

### A. Post‐implant dosimetry

From a clinical perspective, a challenging element of implementing the method presented in this work is calculating the real dose delivered to the patient due to the actual (rather than planned) implant. Since there is inherent seed placement error for PPI procedures, a post‐implant dose calculation is used to assess the potential efficacy of the implant.

It has been standard practice via the recommendations of the Radiation Therapy Oncology Group (RTOG) for this procedure^(^ ^18^ ^,^ ^19^ ^)^ to obtain a one‐month, post‐implant CT of the patient, digitize the seeds, contour the organs and calculate the actual dose delivered. This may not be sufficient in hybrid cases since there are visually identical seeds with different activities — these must be identified. Thus it is necessary to create a one‐to‐one map between the planned seed positions and the actual positions.

This procedure may be complicated by changes in the target volume and shape during the intervening one month between delivery and post‐implant imaging. However, a recent trend^(^ ^20^ ^)^ in PPI procedure is to obtain the post‐implant CT immediately after the implant procedure (rather than after one month). This would mitigate the difficulties in seed matching which stem from debulking and edema. Obtaining the post‐implant CT immediately after the procedure still would not account for the uncertainty inherent in placing seeds, but recent advances in post‐implant seed identification show that a one‐to‐one map can be generated. Brunet‐Benkhoucha et al.^(^ ^21^ ^)^ have shown that with only seven X‐ray images taken with a cone‐beam system subtending a 60° arc, seed detection rates of 96.7%, false negative rates of 3.3%, and false positive rates of 2.7% can be achieved. This is done by locating seeds to 0.4±0.4 mm in 3D space, and resulted in uncertainties in $D_{90}^{\text{Prostate}}$ and $V_{100}^{\text{Prostate}}$ of 1.5% and 0.3%, respectively.

## V. CONCLUSIONS

An optimization algorithm that can generate hybrid brachytherapy plans was developed. Five previously‐treated PPI patients with a range of prostate volumes from 20 to $48\;{\text{cm}}^{3}$ were chosen and reoptimized using the hybrid‐activity PPI optimization. These multi‐activity hybrid plans were equal in quality (as measured by the standard dosimetric indices) to plans with seeds of a single activity. Potential gains achievable by using different radionuclides have recently been explored in the literature; but, since the efficacy of incorporating the BED is still hotly debated in the community, we have focused on the multi‐activity hybrid plans. Despite the expanded search space, optimization times for these studies were still under two minutes on a modern day laptop and can be reduced to below one minute in a clinical setting. With the typical cost of a set of PPI seeds on the order of thousands of dollars, it is possible to reduce the cost of brachytherapy treatments by allowing for easier use of seeds left over from a previous patient or unused due to a cancelled or postponed treatment.

## Footnotes

† We use will use partial activity or fractional activity to describe a seed that has only a fraction of their original activity. Usually this is a seed that was not used immediately after ordering from a vendor (e.g. those left over from a cancelled treatment or from ordering too many seeds for a given implant).

‡ As described in Lessard et al.,^(^ ^8^ ^)^ Lessard and Pouliot,^(^ ^9^ ^)^ Lessard^(^ ^10^ ^)^ and Pouliot et al.,^(^ ^11^ ^)^ the objective function is computed by assessing penalties to doses delivered to organs which are out of the range desired by the medical physicist. The objective function is the main criteria for evaluating plans during the optimization process.

∫ Since the prostate is generally of a regular geometry, this was defined by the intersection of the sagittal‐, coronal‐ and transverse‐bisecting planes.
